# The complete mitochondrial genome of *Oplophorus spinosus* (Brullé, 1839) (Caridea, Oplophoridae)

**DOI:** 10.1080/23802359.2021.1914221

**Published:** 2021-05-12

**Authors:** Xue-Tao Wang, Wen-Ge Shi, Yi-Xuan Li, Xue-Lei Zhang, Qin-Zeng Xu

**Affiliations:** a MNR Key Laboratory of Marine Eco-Environmental Science and Technology, First Institute of Oceanography, Ministry of Natural Resources, Qingdao, China; b Laboratory of Marine Ecology and Environmental Science, Pilot National Laboratory for Marine Science and Technology (Qingdao), Qingdao, China

**Keywords:** Shrimp, Indian Ocean, mitochondrial genome, Oplophorus, phylogeny

## Abstract

We determined the complete mitochondrial genome of *Oplophorus spinosus* with a typical circular structure. The complete mitogenomes of *O. spinosus* was 17,346 bp in length, with 37 genes containing 13 protein-coding genes, 22 tRNAs, two rRNAs, and a confirmed D-loop zone. The GC content of *O. spinosus* was 34.39%. The phylogenetic results showed that *O. spinosus* was most closed to *O. typus*, providing useful mitochondrial information for its further evolutionary and taxonomy study.

*Oplophorus spinosus*, belonging to Caridea, Oplophoridae, has a cosmopolitan distribution including tropical and subtropical waters of the Atlantic, the Pacific, and the Indian Ocean (Sudnik 2018). Oplophoridae which *Oplophorus* belongs to is an important component of deep-pelagic shrimps with abundant quantity and biomass (Vereshchaka et al. 2019). It has cuticular photophores that can make itself bioluminescent (Nowel et al. 1998) and feeds on chaetognaths, juvenile fishes, and other smaller crustaceans (Burdett et al. 2017). Some previous work had a pretty phylogenetic analysis for family Oplophoridae, but the phylogenetic relationship and position of *O.spinosus* were not clear. The complete mitochondrial genome of *O. spinosus* was analyzed to provide new insight about the phylogeny of the genus *Oplophorus.*

The sample was collected from the Indian Ocean (82°56′E, 21°12′S) using Agassiz trawl in Jan. 2019, and the ethanol specimen was stored in Key Laboratory of Marine Eco-Environmental Science and Technology, First Institute of Oceanography, Ministry of Natural Resources (NO. FIO-ECH-DY52DQSP05).

This complete mitochondrial genome was submitted to Genbank and the accession NO. is MW414295. The mitochondrial genome of *O. spinosus* was sequenced on the Illumina HiSeq 2500 Sequencing Platform (Illumina, USA) by Novogene Corporation (Beijing, China). The clean data were assembled using the SPAdes 3.6.1 (Bankevich et al. 2012) and NOVOPlasty (Dierckxsens et al. 2017), then annotated by Geseq (Tillich et al. 2017). The circular mitochondrial DNA of *O. spinosus* is 17,346 bp in length with 34.39% GC content and contains 1 D-loop and 37 genes, including 13 protein-coding genes, two ribosomal RNA genes, and 22 transfer RNA genes.

We constructed phylogenetic analysis based on 13 protein-coding genes of *O. spinosus* and other 12 Caridean shrimps. Meanwhile, *Alpheus inopinatus* and *A. japonicus* were chosen as outgroups. The phylogenetic tree was built by using the Maximum Likelihood method with 1000 bootstrap replicates through IQTREE (Nguyen et al. 2015). The relationship between *O. spinosus* and other Caridean shrimps showed that *O. spinosus* and *O. typus* formed a branch, and then gathered with genus *Caridina* and *Typhlatya* (Figure 1). The results provide additional details for further evolutionary and phylogenetic researches of the genus *Oplophorus.*

**Figure 1.**
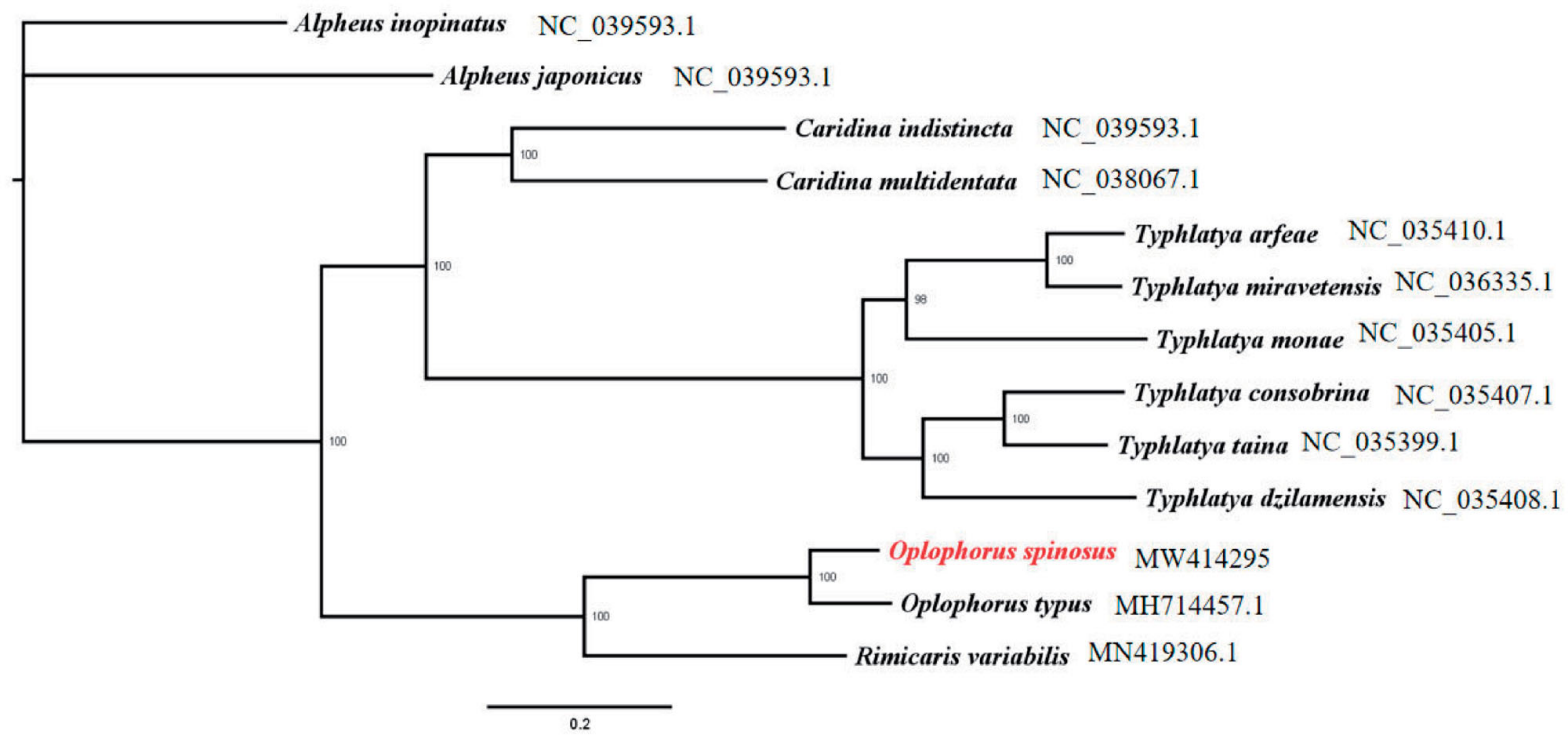
The Maximum Likelihood tree of 13 protein-coding mitochondrial genes in 13 Caridea. Number at each branch, bootstrap probability.

## Data Availability

Mitogenome data supporting this study are openly available in GenBank at https://www.ncbi.nlm.nih.gov/nuccore/MW414295. Associated BioProject, SRA, and BioSample accession numbers are https://www.ncbi.nlm.nih.gov/bioproject/PRJNA687831, https://www.ncbi.nlm.nih.gov/sra/SRR13306937, and SAMN17155447, respectively.
